# Draft Genome Sequence and Description of *Janthinobacterium* sp. Strain CG3, a Psychrotolerant Antarctic Supraglacial Stream Bacterium

**DOI:** 10.1128/genomeA.00960-13

**Published:** 2013-11-21

**Authors:** Heidi Smith, Tatsuya Akiyama, Christine Foreman, Michael Franklin, Tanja Woyke, Hazuki Teshima, Karen Davenport, Hajnalka Daligault, Tracy Erkkila, Lynne Goodwin, Wei Gu, Yan Xu, Patrick Chain

**Affiliations:** Department of Land Resources and Environmental Sciences, Montana State University, Bozeman, Montana, USAa; Department of Microbiology, Montana State University, Bozeman, Montana, USAb; Department of Chemical and Biological Engineering, Montana State University, Bozeman, Montana, USAc; Center for Biofilm Engineering, Montana State University, Bozeman, Montana, USAd; DOE Joint Genome Institute, Walnut Creek, California, USAe; Genome Science Programs, Bioenergy and Biome Science, Los Alamos National Laboratory, Los Alamos, New Mexico, USAf

## Abstract

Here we present the draft genome sequence of *Janthinobacterium* sp. strain CG3, a psychrotolerant non-violacein-producing bacterium that was isolated from the Cotton Glacier supraglacial stream. The genome sequence of this organism will provide insight as to the mechanisms necessary for bacteria to survive in UV-stressed icy environments.

## GENOME ANNOUNCEMENT

Psychrophiles often possess mechanisms to protect themselves from environmental conditions, including extremes of UV radiation, temperature, freeze-thawing events, and desiccation, which cause severe damage to nonadapted organisms (1). Reductions in stratospheric ozone have resulted in formation of the Antarctic ozone hole, causing increased levels of UV B radiation (UVB) to reach the Earth’s surface, specifically affecting Antarctica (2). The highly damaging effects of short-wavelength radiation, UVB, and UV A radiation (UVA) to biological systems have been well documented (3). Short-wave radiation induces damage to a variety of macromolecules (e.g., DNA, RNA, proteins, and lipids) by indirectly increasing the formation of reactive oxygen species (4). The pigment violacein has been shown to possess strong antioxidant properties, protecting cellular lipid membranes from hydroxyl radicals by mitigating peroxidation (5).

*Janthinobacterium* sp. strain CG3 was isolated from a supraglacial fluvial system on the Cotton Glacier, Antarctica (77°07′S, 161°50′E). The bacterial strain was isolated on R2A agar medium incubated at 4°C in the dark for 12 days. The *Janthinobacterium* sp. strain CG3 isolate has characteristics of the genus *Janthinobacterium*, with strictly aerobic, Gram-negative, rod-shaped cells, but does not show evidence of violacein production (6). Additionally, *Janthinobacterium* sp. strain CG3 does not possess genetic evidence of the *vioA* operon, which is responsible for violacein production (7). As *Janthinobacterium* sp. strain CG3 lacks the genetic machinery necessary for violacein production, the genome sequence of this psychrotolerant organism will provide insight into strategies that have been evolved by organisms lacking pigments to survive in UV-stressed polar environments.

The draft genome sequence of *Janthinobacterium* sp. strain CG3 was generated at the DOE Joint Genome Institute (JGI) using Illumina data (8). Short-insert (insert length, 190 bp) and long-insert (insert length, 10,800 bp) paired-end libraries generated 19,156,074 and 21,678,138 reads, respectively. A total of 6,125 Mbp of Illumina data were used to assemble the genome with Allpaths, Velvet, and Parallel Phrap. Initial assemblies generated by Allpaths version 39750 and Velvet version 1.1.05 (9) were computationally shredded into 10-kbp or 1.5-kbp overlapping fake reads, respectively. Overlapping fake reads from Allpaths and two Velvet assemblies were combined with the Illumina long-insert paired-end reads to generate the final assembly by Parallel Phrap version 4.24. Possible misassemblies were corrected with manual editing in Consed (10–12). Sequence gaps were closed by using repeat resolution software and sequencing bridging PCR fragments.

The draft genome sequence of *Janthinobacterium* sp. strain CG3 consists of 71 contigs in 7 scaffolds, with an estimated size of 6.4 Mbp. A total of 5,426 candidate protein-coding genes were predicted, with a total G+C content of 65.5%. The small-subunit rRNA gene sequences had 99% sequence identity to a *Janthinobacterium* sp. isolate from an Arctic glacier (GenBank accession number FM955878). Scaffolds 3, 4, and 7 were circularized with sequences of bridging PCR fragments, suggesting the presence of three plasmids in the *Janthinobacterium* sp. strain CG3 isolate.

### Nucleotide sequence accession numbers.

This whole-genome shotgun project has been deposited at DDBJ/EMBL/GenBank under accession number APFF00000000. The version described in this paper is version APFF01000000.
